# Advancing clinical biochemistry: addressing gaps and driving future innovations

**DOI:** 10.3389/fmed.2025.1521126

**Published:** 2025-04-08

**Authors:** Haiou Cao, Enwa Felix Oghenemaro, Amaliya Latypova, Munthar Kadhim Abosaoda, Gaffar Sarwar Zaman, Anita Devi

**Affiliations:** ^1^Department of Oncology, Heilongjiang Beidahuang Group General Hospital, Harbin, Heilongjiang, China; ^2^Department of Pharmaceutical Microbiology, Faculty of Pharmacy, Delta State University, Abraka, Nigeria; ^3^Department of Medical and Technical Information Technology, Bauman Moscow State Technical University, Moscow, Russia; ^4^Department of Mathematics and Natural Sciences, Gulf University for Science and Technology, Mishref, Kuwait; ^5^College of Pharmacy, The Islamic University, Najaf, Iraq; ^6^College of Pharmacy, The Islamic University of Al Diwaniyah, Al Diwaniyah, Iraq; ^7^College of Pharmacy, The Islamic University of Babylon, Babylon, Iraq; ^8^Department of Clinical Laboratory Science, College of Applied Medical Sciences, King Khalid University, Abha, Saudi Arabia; ^9^Department of Applied Sciences, Chandigarh Engineering College, Chandigarh Group of Colleges-Jhanjeri, Mohali, India

**Keywords:** clinical biochemistry, biomarkers, point-of-care system, artificial intelligence, personalized medicine, mass spectrometry

## Abstract

Modern healthcare depends fundamentally on clinical biochemistry for disease diagnosis and therapeutic guidance. The discipline encounters operational constraints, including sampling inefficiencies, precision limitations, and expansion difficulties. Recent advancements in established technologies, such as mass spectrometry and the development of high-throughput screening and point-of-care technologies, are revolutionizing the industry. Modern biosensor technology and wearable monitors facilitate continuous health tracking, Artificial Intelligence (AI)/machine learning (ML) applications enhance analytical capabilities, generating predictive insights for individualized treatment protocols. However, concerns regarding algorithmic bias, data privacy, lack of transparency in decision-making (“black box” models), and over-reliance on automated systems pose significant challenges that must be addressed for responsible AI integration. However, significant limitations remain—substantial implementation expenses, system incompatibility issues, and information security vulnerabilities intersect with ethical considerations regarding algorithmic fairness and protected health information. Addressing these challenges demands coordinated efforts between clinicians, scientists, and technical specialists. This review discusses current challenges in clinical biochemistry, explicitly addressing the limitations of reference intervals and barriers to implementing innovative biomarkers in medical settings. The discussion evaluates how advanced technologies and multidisciplinary collaboration can overcome these constraints while identifying research priorities to enhance diagnostic precision and accessibility for better healthcare delivery.

## 1 Introduction

Laboratory medicine relies on clinical biochemistry, analyzing biological fluids like blood, urine, and cerebrospinal fluid for disease assessment and treatment guidance. This discipline examines diverse biochemical indicators—metabolites, hormones, electrolytes, and enzymes—generating vital information that shapes medical interventions, enhances patient care and supports precision medicine approaches (1, 2). This field has several applications, including traditional molecular diagnostics and complex biochemical assays. The main objective of clinical biochemistry is to gather precise and dependable statistics, enabling healthcare practitioners to make better-informed decisions, guide treatments, and predict patient outcomes (3).

**FIGURE 1:**
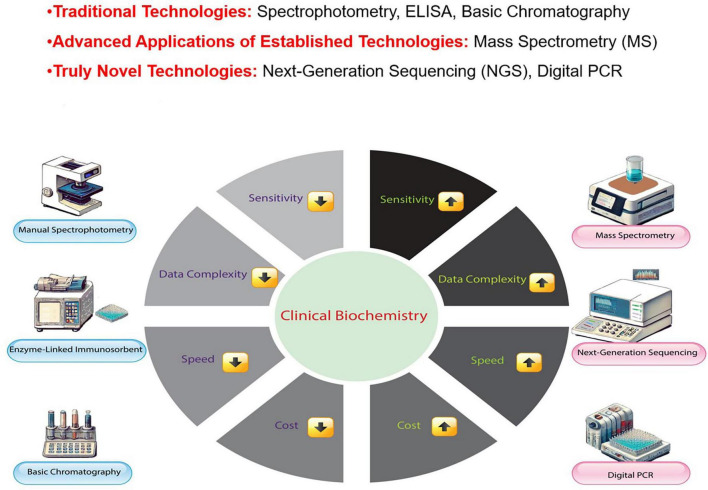
Comparison of traditional and novel technologies in clinical biochemistry. This figure compares Traditional Technologies (manual spectrophotometry, enzyme-linked immunosorbent assays, basic chromatography), Advanced Applications of Established Technologies (mass spectrometry, MS), and Truly Novel Technologies [next-generation sequencing and digital Polymerase Chain Reaction (PCR)] in clinical biochemistry. While MS is a well-established technology, its advanced applications, such as in proteomics and metabolomics, have significantly enhanced its role in modern diagnostic practices, distinguishing it from traditional methodologies.

Biochemical testing is vital in medical assessment, providing critical diagnosis and patient management data. While both speed and precision are essential for timely decision-making, achieving rapid turnaround times sometimes requires a careful balance between the two. In emergency situations, such as acute myocardial infarction or sepsis, rapid preliminary results can guide immediate therapeutic decisions; however, these results are always followed by confirmatory tests using highly accurate methods to ensure diagnostic reliability. In non-emergency cases, accuracy remains paramount, as any compromise could lead to severe consequences, such as a false-negative result for a cancer marker, which may delay critical treatment. Therefore, accuracy is never sacrificed when diagnostic precision is crucial for patient outcomes (4, 5). These analytical results are integral to guiding therapeutic strategies and informing patient care. For example, the early detection of cardiac biomarkers in patients with chest symptoms can be life-saving (6, 7). However, delays or errors in biochemical tests can result in misdiagnosis, incorrect treatment, and potential harm to patients (8). Current methods in clinical biochemistry include immunoassays, chromatography, spectrophotometry, and mass spectrometry (MS), a well-established analytical tool that continues to advance with novel applications and improved instrumentation (9–11). Figure 1 provides a comparative overview of traditional and Novel Technologies in clinical biochemistry, illustrating how advancements in MS, next-generation sequencing, and digital Polymerase Chain Reaction (PCR) have significantly improved sensitivity, speed, and data complexity management, although at a higher cost.

Spectrophotometric analysis is widely adopted due to its cost-effectiveness and operational simplicity. However, sample quality issues, particularly turbid or hemolyzed specimens, may compromise measurement accuracy (9). While chromatographic and MS techniques provide superior analyte specificity and detection sensitivity—especially valuable for therapeutic drug monitoring, toxicological screening, and metabolic profiling—these methods typically require extensive specimen processing, increasing time and resource demands (12). Although immunological assays offer targeted detection capabilities, measurement interference from cross-reactive species and heterophilic antibodies (including HAMA) can affect result reliability (13, 14). Advancing clinical biochemistry requires novel methodological approaches. Recent developments in Micro total analysis system (μTAS) technology, microfluidic platforms, and improved immunological detection systems show promise in addressing current analytical limitations. However, specific technical challenges persist—most notably matrix effects from heterophilic antibodies—emphasizing the continued need for research into more reliable diagnostic methodologies (15). Table 1 compares the principles, advantages, and limitations of the traditional and Novel Technologies in clinical biochemistry.

**TABLE 1:** Comparison of analytical techniques in clinical biochemistry.

Technology	Principle	Advantages	Limitations	Sample volume	Throughput	Cost	References
Spectrophotometry	Absorption of light by analytes in solution	Simple, inexpensive	Limited sensitivity and specificity	Low	High	Low	(16, 17)
Chromatography	Separation of components in a mixture due to their differential distribution between two phases	Highly specific, suitable for complex molecules	Time-consuming; requires extensive sample preparation; high skill required	Medium	Medium	High	(18, 19)
Immunoassays	Antigen-antibody interactions	High specificity, wide analyte range	Cross-reactivity, interference	Low	High	Medium	(20, 21)
Mass spectrometry	Separation and detection of ionized analytes	High sensitivity and specificity; recent advancements enable applications in metabolomics and proteomics	Expensive instrumentation, complex sample preparation	Low	Low–medium	High	(22)
Lab-on-a-Chip (μTAS)	Miniaturized laboratory processes on a chip	Portable, fast, low sample volume required; potential for point-of-care applications	Limited multiplexing capability: some matrix interference issues remain	Very low	High	Medium-high	(23, 24)

Current clinical biochemistry faces persistent methodological challenges despite technological progress. A significant concern is the inadequate reference ranges for many biochemical parameters, particularly among pediatric and geriatric populations (25). While these intervals are crucial for interpreting results, they vary considerably across biological, demographic, and analytical factors. Programs like CALIPER have advanced the development of pediatric reference intervals, though similar initiatives are still needed for other demographic groups (26). The integration of emerging biomarkers into standard clinical protocols poses another critical challenge. Although these novel indicators have the potential for diagnostics and personalized medicine, practical implementation faces obstacles such as analytical standardization, regulatory compliance, and economic viability. Addressing these barriers requires coordinated action among scientific investigators, medical practitioners, regulatory authorities, and health policy experts to optimize patient benefit (27, 28). Clinical biochemistry has the potential for transformative progress by integrating advanced technologies.

Analytical methods at the nanoscopic level enable the detection and characterization of nanoparticles and similar biological structures within human systems, including exosomes, liposomal formations, virus-mimicking particles, and protein clusters (29, 30). These nanometric entities are essential in normal physiology and disease development (30). Extracellular vesicles in the nanometer range, particularly exosomes, serve as diagnostic indicators for early-stage malignancies, neurological disorders, and cardiac pathologies (31). Likewise, nanoscale protein assemblies known as amyloid fibrils are associated with neurodegenerative conditions such as Alzheimer’s and Parkinson’s (32, 33). Using nano-focused analytical techniques provides healthcare professionals and researchers with highly sensitive detection capabilities, facilitating early and accurate disease identification (34, 35). AI systems can improve result interpretation and provide a deep understanding of complex biological phenomena through automated data analysis (36). Bioinformatics integrates molecular biology, genetics, and genomics to characterize biological systems and disease processes (37). This review discusses recent technological advancements in clinical biochemistry, identifies current limitations, and proposes future research directions. By assessing the demand for fast and accurate biochemical tests in diagnostic medicine, exploring available techniques and their shortcomings, and discussing forthcoming tools, we aim to contribute to ongoing efforts to improve the quality of laboratory analyses and maximize the benefits of biomedical research.

## 2 Current gaps in clinical biochemistry

### 2.1 Technological limitations

Despite significant advances, traditional clinical biochemistry techniques such as spectrophotometry, chromatography, and immunoassays are severely limited in their application to modern healthcare. They have insufficient sensitivity, cross-reactivity, delayed results, and high cost, which limit their use in routine testing and precision medicine (38).

#### 2.1.1 Sensitivity and specificity challenges

Spectrophotometry is one of the most commonly used techniques in biochemical testing because it is relatively inexpensive and easy to perform. However, it lacks the sensitivity and specificity to identify biomarkers at low concentrations that are useful for early disease diagnosis. For example, in the diagnosis of cancer and metabolic disorders, studies have shown that MS provides up to 1,000 times lower detection levels for some analytes compared to spectrophotometric methods (39). This difference is important in diseases such as pancreatic cancer, where early detection of markers such as CA19-9 can significantly improve outcomes (40). In addition, immunoassays, which are often used to detect proteins, hormones, and disease markers, can have problems with cross-reactivity and interference from the sample. This can result in false-positive or false-negative results. A review of immunoassay performance in clinical applications found that cross-reactivity rates varied from 0.1% to 3%, with some assays reaching up to 15% in complicated biological samples (41). Such errors can lead to incorrect diagnosis or treatment, particularly in endocrine and infectious disease testing (42).

#### 2.1.2 Speed and reproducibility issues

Turnaround time (TAT) is an important consideration in clinical testing. Spectrophotometric assays typically process samples within 5–10 min, but immunoassays require 2–3 h per run, which can delay clinical decisions (43). Such delays are of particular concern in emergency care settings, such as emergency departments, where rapid test results for cardiac problems (such as troponin levels in myocardial infarction) or indications of sepsis can be life-saving (44). In addition to speed, reproducibility and standardization remain critical in biochemical analysis. Variability in reagents, technique and calibration can affect the reliability of test results. Inter-laboratory variability constitutes a significant consideration within the domain of clinical biochemistry, given its capacity to influence the interpretation of results and the subsequent management of patients (45). While variability exists among different testing sites, accredited laboratories adhere to stringent quality control measures, including internal and external quality assurance programs, to ensure analytical accuracy and precision (46). Standards and guidelines for laboratories, such as those established by the Clinical and Laboratory Standards Institute (CLSI) and the International Federation of Clinical Chemistry and Laboratory Medicine (IFCC), delineate acceptable variability limits for each analyte (47, 48). Consequently, while certain studies have documented higher coefficients of variation for specific analytes in particular settings, these observations might not be universally applicable to all accredited laboratories that adhere to standardized performance criteria (49, 50).

#### 2.1.3 Cost and implementation barriers

Although MS and chromatography-based methods offer better analytical precision, high instrument costs, lengthy sample preparation, and the need for experienced operators are limiting factors in their wider acceptance in routine diagnostics (51). The main barrier is the economic impact on low- and middle-income countries, where access to advanced diagnostic tools is still limited. This makes the use of sophisticated techniques such as next-generation sequencing (NGS) or high-throughput screening (HTS) difficult (52, 53). To address the cost and difficulty of clinical biochemistry, scientists are developing low-cost analytical devices that are both accurate and inexpensive. This will make sophisticated tests more accessible to more people (54, 55). In addition, efforts to increase the automation of laboratory processes aim to improve efficiency, minimize human error, and reduce reliance on highly trained personnel, thereby streamlining operations in both low and high resource settings (55). In addition, strengthening regulatory frameworks and standardization is critical to ensure consistency and reliability across diverse healthcare systems, facilitating the seamless integration of new technologies into routine daily clinical practice (48).

#### 2.1.4 Emerging solutions and future directions

Emerging technologies such as microfluidic-based lab-on-a-chip (μTAS) platforms and point-of-care testing (POCT) devices offer promising ways to overcome technological limitations. Such platforms allow for miniaturized, rapid testing with dramatically reduced sample volumes and are therefore well suited to remote and resource-limited settings (56, 57). However, limitations such as lack of multiplexing capability, standardization, and regulatory approval processes still need to be addressed before widespread clinical use (58). AI and ML in clinical biochemistry can address these issues technologically. AI-based data analysis can make diagnoses more accurate, improve test consistency, and streamline laboratory procedures, thereby reducing errors and variability (58, 59). Collaboration among researchers, clinicians, and industry stakeholders is needed to translate these technological advances into daily clinical practice (57).

### 2.2 Clinical challenges

Clinical biochemistry encounters various challenges that significantly influence patient care and health outcomes. One critical issue is the prolonged TAT for essential biochemical tests, especially in acute care settings where timely interventions are crucial (1). For instance, delays in cardiac biomarker results, such as troponin tests, can negatively impact patient outcomes in suspected acute myocardial infarction cases (60). Similarly, the lack of sensitive and specific biomarkers for early disease detection, including pancreatic cancer, with a 5-year survival rate of only 9%, is a significant obstacle to timely diagnosis and improved prognosis (61). The absence of definitive early biomarkers for Alzheimer’s disease further impedes timely intervention, limiting the ability to detect the disease before symptoms manifest (62). Pregnancy-related physiological changes necessitate the establishment of specialized reference intervals for biochemical parameters. For example, The diagnosis and management of pregnancy complications necessitates the assessment of hormone levels, including thyroid-stimulating hormone (TSH) and thyroid hormones (free T4 and total T4) (63, 64). The literature has proposed various reference values for TSH, aiming to enhance diagnostic accuracy by considering the direct effects of pregnancy on thyroid hormone metabolism (65, 66). However, in standard clinical practice, TSH testing is often accompanied by additional T4 hormone assessments, as these provide a more precise assessment of maternal thyroid function and fetal wellbeing (67, 68).

Standardizing biochemical tests across laboratories and healthcare systems is crucial for ensuring consistent and reliable results for patient care. The International Federation of Clinical Chemistry and Laboratory Medicine (IFCC) is involved in global initiatives to harmonize test results (69, 70). Challenges in achieving this standardization include variations in methodologies, reagents, and calibration practices, which can lead to discrepancies in results and affect patient diagnosis and treatment (48). Metrological traceability to higher-order references is essential for the equivalence of the measurement results. However, its correct implementation is hindered by the complexity of the task and the need for improved collaboration among stakeholders, including reference providers, *in vitro* Diagnostics (IVD) manufacturers, and laboratory professionals (48, 69). Enhancing education, training, and regulatory alignment are crucial steps toward overcoming these challenges and achieving global harmonization of biochemical tests to improve patient outcomes (69). Integrating biochemical data with other clinical information sources, such as genomic data and imaging studies, is critical for advancing personalized medicine.

Electronic health records (EHRs) are pivotal for improving data accessibility; however, effectively combining diverse datasets for comprehensive patient assessment remains a significant challenge (71, 72). Resource limitations significantly impact the adoption of new biochemical technologies, particularly in low-income and middle-income countries. Advanced techniques, such as MS and NGS, while offering superior analytical capabilities, require substantial financial investment and specialized expertise. A survey by the World Health Organization found that only 65% of low-income countries had access to elemental biochemistry analyzers, let alone advanced technologies (73). Modern research areas like metabolomics and proteomics generate extensive datasets that challenge traditional laboratory practices. These disciplines demand advanced data interpretation and clinical implementation expertise, creating significant training requirements for laboratory personnel. Integrating complex molecular information into practical healthcare requires specialized knowledge to translate biological profiles into actionable clinical insights (74–76). These advanced methods require specialized training programs to ensure staff proficiency and clear guidelines for interpreting results to translate technological advancements into improved patient care (77). Bioinformatics is crucial for analyzing chemical information acquired through MS for clinical analysis, highlighting the importance of data interpretation and integration in complex biological samples (74). Furthermore, the application of AI in interpreting omics data for disease diagnosis and developing network atlases to analyze biochemical relationships between organs demonstrates the evolving landscape of clinical bioinformatics (74). As the field progresses, it has become essential to establish comprehensive training programs and guidelines to maximize the benefits of these advanced technologies to enhance patient outcomes and clinical decision-making.

Addressing these complex challenges requires a collaborative approach that involves clinicians, biochemists, and bioinformaticians. Clinical biochemistry can significantly improve patient outcomes and enhance healthcare delivery by developing rapid point-of-care diagnostics, identifying and validating new biomarkers, establishing reference intervals specific to different populations, and implementing effective data management systems.

### 2.3 Data management issues

Clinical biochemistry has made considerable advancements; however, it still faces challenges in utilizing large amounts of data. Restrictions in data management and analysis impede efforts to enhance patient care. Data integration and interoperability are significant obstacles because incompatible data formats and technologies create silos that hinder patient records and research (1). The extended amount and complexity of biochemical assay data overwhelm conventional methods for data analysis, such as manual interpretation, essential statistical tools, and spreadsheet-based approaches, which are often inadequate for processing large, multidimensional datasets generated by modern assays (78, 79). This wastes crucial information. It is crucial to understand diseases and treatments (80). Robust data management systems and bioinformatics platforms are vital answers. These solutions must incorporate diverse types of data. It incorporates EHRs, laboratory information systems, and omics data (81). Standardized data formats and seamless data communication are crucial for breaking down information silos (81). Overcoming these challenges will unlock the full potential of big data in clinical biochemistry, enabling better data integration and analysis, which will drive the development of tailored medicine. This, in turn, facilitates automated patient data analysis, risk prediction, and individualized therapy (82, 83). Bioinformatic technologies with actionable information will empower clinicians and improve patient outcomes and advancements in this field. Although challenges still exist, the evolving world of data management and analysis presents a significant opportunity. These advancements will usher in an era of personalized medicine characterized by accurate diagnosis, effective therapies, and enhanced patient care (84).

## 3 Novel technologies in clinical biochemistry

### 3.1 HTS and automation

**FIGURE 2:**
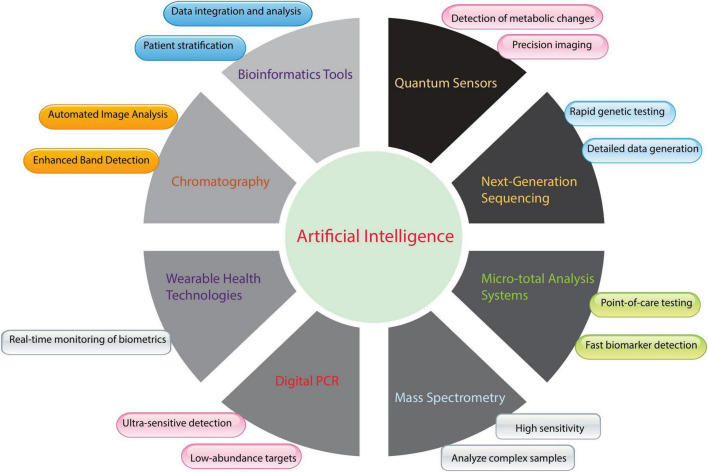
Integration of artificial intelligence (AI) with emerging and established biochemical tools in clinical biochemistry. Figure 2 shows AI’s transformative role in clinical biochemistry instrumentation and methodologies. Machine learning algorithms enhance conventional analytical techniques, such as chromatographic separation and mass spectrometric analysis, by providing automated visual processing, improved signal detection, heightened analytical sensitivity, and sophisticated interpretation of complex samples. AI integration extends to advanced technologies, including rapid genetic analysis through next-generation sequencing (NGS), continuous physiological monitoring via wearable devices, and metabolic assessment using quantum sensing platforms. Furthermore, AI capabilities support bioinformatics applications for comprehensive data synthesis and patient classification, enable precise quantification through digital Polymerase Chain Reaction (PCR), and support miniaturized analytical systems for rapid diagnostic testing. This technological convergence advances laboratory medicine by optimizing analytical performance, operational efficiency, and diagnostic utility.

Medical laboratories use computerized equipment to detect abnormalities. This equipment has revolutionized clinical biochemistry by making tests significantly faster and more accurate. These machines can process numerous samples daily, reduce manual work, and expedite diagnoses. Automated analyzers eliminate human errors and yield reliable results (85). Consequently, microfluidics and lab-on-a-chip technologies have transformed biochemical testing. These models combine laboratory activities into a single microchip, enabling the rapid screening of diverse samples with fewer reagents (86). Lab-on-a-chip devices conduct complex biomedical tests on-site and deliver swift results. As shown in Figure 2, AI plays a pivotal role in integrating various innovative technologies, such as bioinformatics tools, quantum sensors, NGS, and micrototal analysis systems. These technologies and advancements in tools like MS are transforming traditional clinical biochemistry practices.

### 3.2 Advanced analytical techniques

Mass spectrometry is a well-established and indispensable tool in clinical biochemistry due to its unparalleled ability to detect and measure biomolecules with high sensitivity and specificity. Recent advancements in MS, such as improved resolution, miniaturization, and novel proteomics and metabolomics applications, further enhance its utility in clinical diagnostics (87, 88).

Nuclear Magnetic Resonance (NMR) spectroscopy, a well-established analytical technology, plays a valuable role in clinical biochemistry by providing detailed insights into biomolecules, including their structure and behavior. Its non-destructive nature allows for repeated measurements, making it particularly useful for longitudinal studies. Advances in NMR applications, such as metabolomics and structural biology, have expanded its utility in understanding disease mechanisms and identifying potential drug targets (89). NGS has changed genomic and metabolic profiling. It provides unmatched data depth and breadth. NGS examines genomic variants, helps to identify disease-causing mutations, and enables personalized medicine. Rapid genome sequencing has opened new avenues of research and diagnosis (90).

Researchers have made critical strategic changes in clinical biochemistry by introducing new methods for determining hemoglobin A1c (HbA1c) in diabetes control (91, 92). The HbA1c biomarker is important for assessing long-term glycemic control, as it indicates blood glucose levels over the preceding 2–3 months (93). Traditional HbA1c methods like high-performance liquid chromatography (HPLC) are standard, but these methods have been constrained by interference from hemoglobin variants and sample prep time (91, 94).

In recent years, the development of high-throughput and automated capillary electrophoresis and MS has led to significant advancements in reducing analytical variability (95). For example, HPLC is the most effective method for separating HbA1c from other hemoglobin variants that have been demonstrated to interfere with blood tests (96). This technique has been demonstrated to be effective in populations affected by hemoglobinopathies (97). In addition, matrix-assisted laser desorption/ionization-time of flight (MALDI-TOF) MS is advanced as the most sensitive technique of quantification and method for detecting HbA1c while eliminating modified hemoglobin species (38, 98).

Point-of-care (POC) testing has been further advanced by providing compact immunoassay-based analyzers that can perform the tests within minutes (99). The Abbott Afinion 2 and Siemens DCA Vantage devices are among those that have proven promising results (99, 100). They are a foundation for new methods alongside traditional laboratory-based techniques that allow real-time diabetes management in clinical and primary settings (101). Integrating these new analytical techniques enhances diagnostic accuracy, allowing for better patient monitoring and personalized treatment strategies (102).

### 3.3 Point-of-care testing (POCT)

Point-of-care testing has transformed healthcare delivery through accessible diagnostic capabilities at patient treatment sites. These compact, user-oriented platforms facilitate immediate result generation, enabling rapid clinical decision-making and minimizing follow-up requirements (103). POCT implementation demonstrates utility in geographically remote or resource-constrained environments, enhancing healthcare accessibility and intervention timeliness (104). Treatment modalities have evolved significantly, particularly in diabetic care protocols (105). The introduction of CGM technology in the early 2000s marked a shift from conventional capillary blood sampling methods, allowing for continuous glucose level monitoring (106). Subsequent advancements, including pre-calibrated sensing elements, improved measurement accuracy, increased sensor durability, and compatibility with automated insulin delivery systems, have fundamentally reshaped diabetes management strategies (107). These developments enable precise insulin dosing while minimizing patient intervention, leading to better glycemic control and improved patient wellbeing (108). POCT in infectious disorders enables rapid diagnosis and appropriate treatment, limiting disease transmission and improving patient outcomes (109, 110). POCT devices are user-friendly, portable, and provide quick results, revolutionizing healthcare delivery by facilitating faster diagnosis, reducing costs, and increasing patient satisfaction (103). Moreover, integrating rapid diagnostic technologies such as molecular diagnostics and handheld PCR machines expands healthcare services to remote areas and ensures prompt pathogen identification for timely treatment initiation in resource-limited settings (109). The bedside application of point-of-care ultrasound (POCUS) significantly enhances diagnostic accuracy in infectious disorders, aids in the antibiotic selection, identifies sources of infection, and guides non-surgical invasive procedures that impact patient care and treatment outcomes (110). These findings highlight the transformative power of POCT in healthcare delivery and effective management of infectious diseases.

POCT devices utilize various technologies, including biosensors, lateral flow tests, and microfluidic devices. Biosensors such as glucose and electrochemical sensors employ biological recognition components to detect and quantify specific analytes (111, 112). Lateral flow assays (LFAs) are widely used for rapid diagnostic testing due to their simplicity and affordability (113). Traditional LFAs rely on the capillary flow of the sample through a membrane strip containing immobilized antibodies or other recognition features, such as nitrocellulose membranes (114). Innovations in LFA technology incorporate advanced design elements to optimize performance. The integration of SS Mem technology uses specialized surface architecture to enhance capillary action, facilitating faster sample migration and uniform distribution while minimizing surface retention (115). These modifications reduce analysis times, decrease background interference, and improve analytical sensitivity. Concurrent developments in microfluidic platforms enable precise fluid control through engineered channels, optimizing reagent interactions while conserving specimen volume (116). This technological convergence enhances LFA capabilities for detecting trace analytes, broadens quantitative measurement ranges, and expands their utility in decentralized diagnostic applications across various pathological conditions (117). Microfluidic devices, or lab-on-a-chip systems, consolidate multiple laboratory operations on a small substrate, enabling complex analysis with minimal sample quantities (118). These developments address the limitations of traditional LFAs and offer a viable alternative for improving their performance and reliability in various applications, such as food verification and POCT. Although POCT offers numerous benefits, it also presents significant drawbacks and limitations. Quality control and standardization are crucial for obtaining accurate and reliable test results, particularly in decentralized settings (117, 119). Operator training and adherence to correct procedures are essential for reducing errors and ensuring the accurate interpretation of test results. Furthermore, POCT data must be integrated with EHRs and laboratory information systems (LIS) to ensure seamless data management and continuity of care (120, 121).

Strategic approaches to addressing these constraints include implementing standardized quality measures, developing structured training protocols, and enhancing data management system integration (122, 123). Oversight bodies maintain stringent standards for POCT device validation through comprehensive evaluation protocols. The FDA’s regulatory framework in the United States categorizes these instruments according to potential risk, with most POCT systems designated as Class II devices necessitating 510(k) submission documentation demonstrating comparability to previously sanctioned instruments (124, 125). FDA guidelines establish specific criteria for analytical performance validation, emphasizing measurement reliability and operational accessibility across various testing environments. CLIA regulations further mandate simplified testing procedures and minimal risk potential for waived assessments (126, 127).

The European regulatory landscape underwent a significant transformation with the implementation of IVDR (EU 2017/746), superseding the previous IVDD framework. This updated regulation implements enhanced requirements for clinical validation, performance verification, and continuous monitoring protocols. The IVDR framework introduces stratified risk assessment, mandating third-party conformity evaluation for high risk diagnostic platforms. Manufacturers must provide comprehensive technical documentation while ensuring adherence to GSPR standards. Both regulatory frameworks prioritize patient safety, analytical precision, and operational reliability in POC applications, establishing robust quality assurance mechanisms for market authorization (128–130).

Future developments in POCT anticipate advancement in multiplexed analytical capabilities, enabling concurrent detection of multiple parameters from individual samples (131). Integration with wearable devices and mHealth platforms will enhance continuous monitoring capabilities and data acquisition. Additionally, incorporating AI and ML methodologies will optimize result interpretation and clinical decision support, advancing diagnostic efficiency (132).

### 3.4 Biosensors and wearable technologies

Integrating biosensor technology and wearable devices has revolutionized clinical biochemistry through continuous, real-time physiological monitoring capabilities, enhancing personalized medical care approaches (133). While POCT systems deliver rapid diagnostic information at patient sites for immediate clinical decisions, biosensors, and wearable platforms facilitate sustained, minimally invasive parameter tracking. POCT applications primarily serve separate diagnostic purposes, whereas biosensor and wearable systems enable extended health surveillance and remote patient monitoring protocols (134, 135).

Biosensor advancement optimizes detection sensitivity and minimizes specimen requirements for sustained biological monitoring applications. These platforms combine biological recognition components with signal transduction elements to generate quantifiable measurements from biochemical interactions. Electrochemical biosensor systems, exemplified in glucose monitoring applications, quantify enzymatic reaction-generated electrical signals correlating with glucose levels (136, 137). CGM systems represent significant technological progress, providing dynamic glucose measurements that enhance diabetic care management (138). Advanced biosensor platforms expand monitoring capabilities beyond glycemic control, including cardiac markers, ionic parameters, and additional clinical indicators. Optical biosensor technologies utilize photometric alterations induced by biological processes, enabling non-invasive monitoring of electrolytes in dermal fluid and interstitial compartments. Wearable platforms incorporate biosensor elements within smart devices and fitness monitoring systems, facilitating data transmission to mobile health platforms and Internet of Things (IoT) networks, thus enabling remote patient monitoring and therapeutic customization (139–141). Current biosensor and wearable technologies face significant implementation challenges, including measurement precision, biological compatibility, information security, and patient adherence. Regulatory bodies, such as ISO and FDA, provide comprehensive guidelines to ensure these devices meet established safety standards, performance metrics, and quality parameters (142, 143). Emerging developments in this field include minimally invasive sensing platforms, integrated multi-parameter monitoring systems, and the convergence of biosensor technology with AI for dynamic data processing and predictive analytics. Continuous measurement of biochemical indicators and comprehensive patient information allows AI systems to identify patterns, predict health risks, and generate individualized recommendations (144). Cross-disciplinary research initiatives promote technological advancements and clinical implementation, enhancing disease detection capabilities and therapeutic outcomes (145). Table 2 provides an overview of emerging technological applications in clinical biochemistry, focusing on biosensors and wearable innovations.

**TABLE 2:** Applications of novel technologies in clinical biochemistry.

Technology	Applications
Mass spectrometry	Proteomics, metabolomics, drug monitoring, toxicology
Next-generation sequencing	Genetic testing, cancer genomics, microbial identification
Microfluidics/μTAS	POCT, HTS, biomarker analysis
Biosensors	Continuous glucose monitoring, cardiac monitoring, disease biomarker detection
Artificial intelligence	Predictive diagnostics, personalized medicine, data integration, workflow optimization

### 3.5 AI and ML

Artificial intelligence and ML have revolutionized clinical biochemistry. They transformed data processing and interpretation (146). These technologies use advanced AI algorithms to dissect intricate datasets precisely using modern analytical techniques. Their analysis revealed patterns, correlations, and insights beyond human expertise. This has proven beneficial in metabolomics, proteomics, and genomics, where large and complex datasets challenge traditional methods (147).

Implementing AI-driven systems in clinical biochemistry facilitates personalized diagnostic modeling through advanced algorithmic analysis. These platforms enable refined risk prediction, early disease detection, and metabolic-based patient stratification by processing diverse patient datasets, including clinical observations, biochemical measurements, and molecular signatures (148, 149). This personalized approach enhances diagnostic accuracy and timeliness, supporting early disease recognition and intervention. The resulting improvement in diagnostic capabilities allows clinicians to implement targeted assessment strategies, ultimately optimizing patient care pathways (150, 151). Deep learning architectures, particularly those using neural network frameworks, excel in biomarker discovery, disease diagnostics, and therapeutic target identification (152, 153). Variants of neural networks, specifically Convolutional Neural Networks (CNNs) and Deep Neural Networks (DNNs), outperform conventional analytical methods in early cancer detection using proteomic datasets (154). Research has also highlighted the effectiveness of deep learning systems, including 2D CNN architectures, in accurately predicting anticancer peptide sequences with improved sensitivity and specificity (155). Recent technological advancements include MTS’s novel AI-enhanced pathology platform, designed to streamline laboratory diagnostic processes. This system employs sophisticated ML algorithms for histopathological image analysis, enabling automated identification of pathological features, such as malignant tissue patterns, with high diagnostic precision. Integrating advanced image recognition with predictive analytics enhances workflow efficiency, reduces diagnostic uncertainty, and improves disease classification (156, 157). Natural Language Processing (NLP) techniques have been instrumental in extracting valuable information from unstructured clinical notes and literature, enabling knowledge discovery and the development of decision support systems in healthcare (158).

Artificial intelligence integration with LIS transforms operational paradigms through enhanced workflow automation and data management capabilities. AI-enhanced LIS platforms streamline fundamental laboratory processes, including specimen management, testing coordination, and documentation protocols, significantly reducing manual administrative demands (159). Incorporating AI algorithms within LIS frameworks enables predictive equipment maintenance by analyzing operational metrics and identifying potential system failures early, minimizing operational disruptions and maintenance costs. Advanced analytical capabilities further enhance LIS functionality by identifying operational constraints, detecting result irregularities, and generating strategic recommendations for process improvement. Furthermore, AI-augmented LIS platforms promote system interoperability by integrating diverse analytical instrumentation and clinical platforms, facilitating seamless information exchange across healthcare networks (160). This comprehensive data integration ensures immediate accessibility of laboratory results for clinical personnel, enhancing collaborative care delivery and treatment outcomes.

Artificial Intelligence applications in laboratory medicine optimize the entire workflow, support decision-making, and improve diagnostic outcomes, with AI models showing exceptional predictive capabilities for early sepsis detection and prognostication (161). Furthermore, AI-driven clinical decision platforms enhance diagnostic capabilities by comprehensively analyzing biochemical parameters and identifying significant correlations and patterns denoting various pathological conditions, including metabolic disorders, endocrine dysfunction, and malignancies (162, 163). These systems automate the identification of biochemical abnormalities, generate potential diagnostic considerations, and establish case prioritization protocols (163). In cardiac diagnostics, AI algorithms evaluate temporal troponin variations, assess myocardial injury risk profiles, and recommend appropriate clinical responses. These platforms provide tailored, evidence-based diagnostic guidance by dynamically integrating individualized patient metrics and contemporary clinical protocols. This technological enhancement reduces cognitive demands on practitioners, improves diagnostic accuracy, optimizes clinical workflows, and facilitates better therapeutic outcomes (164, 165).

## 4 Challenges and limitations of adopting novel technologies

While emerging technologies have the potential to advance clinical biochemistry significantly, their implementation is not without challenges and limitations. These include regulatory difficulties, integration with current systems, data management issues, and cost considerations.

### 4.1 Regulatory challenges

In clinical biochemistry, adhering to existing rules when developing new technologies is essential to ensure safety, efficacy, and ethical standards (166). Regulatory bodies like the FDA, EMA, and CLSI are vital for assessing and approving different technologies and prioritizing safety, efficacy, and quality. Procedures can vary because of the unique features and challenges of technologies such as *in vitro* diagnostic equipment, biosensors, and AI-based software (167, 168). The regulation of new devices, such as ML and AI, involves algorithm transparency, data privacy, and adapting evolving models. Partnerships between organizations, such as the Global Harmonization Task Force (GHTF) and the International Medical Device Regulators Forum (IMDRF), are necessary to address these challenges on a global scale (169). Using real-world evidence, medical devices must be monitored after market authorization to ensure proper functioning, safety, and quality (170). Compliance with regulatory requirements, sound risk management strategies, and quality assurance standards are fundamental to patient safety and healthcare innovation (171). Involving stakeholders, promoting open communication channels, and fostering active cooperation are essential for regulatory compliance and healthcare product safety. Access to innovation promptly depends on navigating the regulatory environment, which, if not properly overseen, could hinder adoption or compromise patient safety (171). To address these challenges, developers, regulators, and healthcare professionals must work together to ensure that standards are appropriate for encouraging innovation while safeguarding patient wellbeing (172).

### 4.2 Costs

Adopting new technologies in clinical biochemistry, such as POCT devices and wearable biosensors, requires substantial initial investments in expensive instruments and advanced software that incorporate AI/ML algorithms (135, 173). Although these initial costs are significant, the long-term savings and economic benefits are considerable. These include enhanced efficiency, reduced TAT, better patient outcomes, and lower healthcare costs (135). These technologies facilitate the early detection of diseases, prevent costly complications, and significantly save healthcare systems (135). It is essential to conduct comprehensive cost-effectiveness analyses to justify such investments. These analyses offer a holistic economic value assessment and help prioritize technologies with the most significant long-term benefits (174). Strategies such as equipment sharing, outsourcing analyses, and leveraging cloud-based solutions can optimize costs. Investigating reimbursement opportunities and establishing partnerships can help mitigate the financial burden (135). Ultimately, the economic impact goes beyond individual providers and contributes to systemic cost savings, improved patient access to advanced diagnostics, and enhanced healthcare delivery (174).

### 4.3 Training

Laboratory professionals and doctors should receive extensive training to effectively implement advanced clinical biochemistry technologies. These training programs should cover theoretical knowledge, practical skills, data analysis, and the interpretation of results from cutting-edge tools, such as mass spectrometers and NGS platforms (175). Training can be provided through workshops, online courses, simulation-based learning, and mentorship programs. Manufacturers and vendors typically offer initial and ongoing training on their equipment. Specialized courses provided by academic institutions and professional groups can help bridge knowledge gaps (176). Interdisciplinary training involving laboratory personnel and doctors can improve communication regarding the capabilities and limitations of new technologies. This will make laboratory data more meaningful for clinical decision-making, leading to better collaboration between the two sides (177). Continuous education is crucial for professionals to keep up with the rapid pace of modern innovation. This ensures they maintain high-performance levels by consistently updating their knowledge base through retraining activities and assessing their capabilities, such as competency exams. Furthermore, training should address data privacy, ethics, and bioinformatics concerns because new technologies generate increasingly complex datasets that require analysis (178, 179). Flexible learning options and prioritizing critical skills can mitigate challenges in implementing training programs, such as time constraints and budget limitations. International collaborations and exchanges can provide additional knowledge-sharing opportunities and skill development (180). Practical assessment of training outcomes is crucial to ensure that programs meet their objectives. This can be achieved through practical evaluations, knowledge tests, and monitoring of laboratory performance metrics. By prioritizing comprehensive and ongoing training, clinical biochemistry laboratories can maximize the benefits of innovative technologies, leading to improved patient care and outcomes (181, 182).

### 4.4 Ethical and social implications

Integrating modern technology into clinical biochemistry raises significant ethical and societal concerns. AI algorithms must be transparent, neutral, and regularly audited to ensure fair healthcare delivery (183). Privacy concerns regarding the use of personal data in analytics require robust data protection rules and compliance with regulations such as the General Data Protection Regulation (GDPR) (184). In healthcare, incorporating big data and AI-driven analysis necessitates evolving informed consent processes to address complexities and potential inequities (185, 186). Ethical issues surrounding genetic testing and personalized medicine, including genetic privacy and the right not to know, must be carefully addressed (187). Striking a delicate balance between individual privacy and the public health benefits of data sharing presents ongoing challenges that must be effectively managed (188). To address these complex concerns, healthcare workers should receive ongoing ethical training and be supervised by ethics committees to ensure proper management of the ethical implications of these technologies (189).

### 4.5 Risks and ethical considerations of AI in clinical biochemistry

Artificial intelligence has great potential to increase accuracy and efficiency in clinical biochemistry (190). However, they also create important dangers and moral difficulties. One of the most significant concerns is algorithmic bias, which refers to a model’s training on datasets not fully representative of the patient population (191). Patients can be diagnosed and treated differently. Studies have found that AI-based diagnostic tools may show biases that can impair patients who are already under-represented in the datasets used for training. In addition, “black box” AI models do not offer the transparency needed to allow clinicians to understand how they develop their results. Explainability holds great importance in the context of clinical responsibility (192, 193).

Moreover, automation should not replace human expertise but rather complement it. Overreliance on AI-driven systems without adequate human oversight can introduce risks, particularly in cases in which AI misinterpretations lead to incorrect diagnoses or inappropriate treatment choices (194). Regulatory bodies such as the FDA and EMA emphasize critically validating and monitoring AI applications in healthcare for their reliability and safety before their various uses (195). AI models are trained on large datasets that sometimes include sensitive patient information. Therefore, severe regulations must be imposed to ensure data privacy and security. The GDPR and the Health Insurance Portability and Accountability Act (HIPAA) have been discussed (195). A human-in-the-loop (HITL) approach is recommended, in which AI only assists clinicians in decision-making but does not replace them (196). Future improvements could focus on transparent and interpretable AI models, better dataset diversity, and solid regulations ensuring that AI fits ethical and clinical norms. Clinical biochemistry can benefit from AI while providing care for patients and professional supervision.

## 5 Bridging the gaps with novel technologies

### 5.1 Improved sensitivity and specificity

Technological advancements in clinical biochemistry have significantly enhanced the sensitivity and specificity of diagnostic tests. These improvements allow for the precise detection and quantification of biomarkers even at extremely low concentrations, aiding in the early diagnosis of diseases. High sensitivity assays, such as immunoassays and mass spectrometry-based techniques, have revolutionized the detection of cardiac markers, tumor markers, and infectious agents (197, 198). The improved sensitivity of these technologies reduces false-negative results, ensuring that patients with conditions are accurately identified. Similarly, enhanced specificity minimizes false-positive results, reducing unnecessary follow-up tests and interventions. These advances are particularly significant in domains such as oncology, where early and accurate detection of biomarkers can directly impact treatment success and patient outcomes (199, 200). Integrating AI and ML further enhances diagnostic accuracy by analyzing complex datasets and identifying subtle patterns within biochemical data. These technologies improve the reliability of test results and reduce variability associated with manual interpretation (201). Combining high sensitivity assays with advanced computational tools allows clinical laboratories to achieve unprecedented diagnostic precision across various diseases (201, 202).

### 5.2 Timeliness and efficiency

Clinical biochemistry advances have markedly enhanced diagnostic efficiency and timeliness. Laboratory automation streamlines operational workflows, minimizes manual processing errors, and reduces result TAT (203). POCT systems deliver immediate diagnostic results in bedside and outpatient environments, eliminating traditional testing delays related to specimen transport and centralized analysis (204). These technological developments minimize intervals between specimen collection and result availability, proving essential during emergencies like cardiac episodes or sepsis, where prompt intervention directly impacts survival rates (205). Such rapid analytical capabilities enhance clinical outcomes while optimizing healthcare delivery systems. LIS integration further amplifies operational efficiency through automated data processing and enhanced laboratory-clinical communication protocols (206).

### 5.3 Personalized medicine

Modern clinical biochemistry technologies serve as fundamental drivers in personalized medicine advancement. These platforms enable precise biomarker detection, facilitating individually tailored therapeutic approaches (207). Advanced analytical methods, including NGS and MS, comprehensively analyze genetic, proteomic, and metabolomic profiles, revealing disease mechanisms and patient-specific variations (208). Patient-specific medicine optimizes treatment effectiveness while minimizing adverse reactions through molecular profile-guided therapy selection. This methodology demonstrates value in oncology, where targeted interventions address tumor-specific genetic alterations (209). Enhanced capability to predict therapeutic responses and disease trajectories enables evidence-based clinical decisions, advancing care quality and patient outcomes (210). AI integration and large-scale data analytics strengthen personalized medicine by identifying complex patterns within extensive clinical datasets, optimizing treatment protocols, and improving response prediction accuracy. These innovations transform healthcare delivery by transitioning from standardized protocols toward individualized therapeutic approaches.

### 5.4 Data integration and management

Data integration and management are crucial for utilizing innovative technologies in clinical biochemistry. Advanced bioinformatics tools enhance data interoperability, facilitating the seamless integration of diverse data sources and platforms (211). This information is vital to comprehensive patient records and collaborative research. AI is pivotal for integrating multi-omics data, such as genomics, proteomics, and metabolomics. AI algorithms can rapidly analyze complex datasets and identify patterns and correlations beyond human capabilities (81). A thorough understanding of patient health and disease is essential in precision medicine. AI-driven predictive analytics can identify patients at high risk of requiring early intervention (212). Moreover, implementing AI in LIS optimizes data and improves diagnosis, efficiency, and personalized medicine (213).

## 6 Case studies and current applications

### 6.1 Successful integration of novel technologies

Introducing new technology has revolutionized clinical biochemistry and improved diagnosis and operational efficiency. Micro total analysis systems (μTAS) paired with electrochemistry enable the accurate and rapid assessment of biomarkers, which are crucial for early detection and treatment. Integrated diagnostic systems that combine biochemical and molecular diagnostics offer a comprehensive understanding of patient cases, allowing for targeted and customized therapeutic approaches. One way in which these innovations have improved patient care is by expediting the diagnosis and initiation of treatment, particularly in acute therapy situations. This is achieved by streamlining laboratory processes through upgraded biochemical tests, reducing TAT, enhancing diagnostic capabilities, improving patient outcomes, and optimizing resource use (214, 215).

### 6.2 Comparative analysis of traditional vs. novel technologies

The cost-effectiveness of innovative technologies is crucial to their widespread acceptance in clinical biochemistry. Although the initial investment may be substantial, long-term benefits usually outweigh these costs (216). For instance, while μTAS devices can be costly to implement, they reduce reagent usage and the workforce, thus lowering operational costs and increasing throughput. Furthermore, these technologies greatly enhance clinical outcomes (217). In contrast, traditional biochemical diagnostics, although reliable, often lack the necessary sensitivity and specificity for early identification of illness. Advanced diagnostic platforms, such as those that incorporate immunodetection, can simultaneously identify multiple biomarkers and provide a comprehensive disease profile. This precision is particularly significant in diseases such as osteoarthritis, where early identification can significantly improve patient outcomes (218).

## 7 Future directions and research potential

### 7.1 Future trends in clinical biochemistry

Clinical biochemistry’s trajectory aims to integrate and refine modern technological platforms to optimize diagnostic precision, healthcare accessibility, and personalized patient care. Advancing beyond current methodologies, including MS analysis, NGS technologies, biosensor applications, and AI systems, the field anticipates a shift toward automated processes, continuous monitoring, and comprehensive analytical platforms. These innovations facilitate faster diagnostics with improved accuracy, ultimately enhancing therapeutic outcomes (144). Emerging developments include high-throughput analytical systems designed to process larger sample volumes with greater efficiency and shorter processing times (219). Laboratory workflow enhancements through automation and system miniaturization will increase diagnostic accessibility across various healthcare settings. Specifically, advanced microfluidic systems and integrated diagnostic platforms are expected to provide cost-effective, portable analytical solutions for centralized and distributed testing environments. Integrating multi-omics data—encompassing genomic, proteomic, and metabolomic analyses—significantly advances clinical applications (220). This comprehensive approach, enhanced by AI and ML, enables a deeper understanding of pathological mechanisms, facilitating tailored therapeutic strategies. AI applications will continue improving predictive diagnostics through pattern recognition in complex datasets, enabling early pathology detection and refined risk assessment protocols. Biosensor platforms and wearable technologies are set to become essential components of routine healthcare delivery, allowing for sustained, non-invasive monitoring of biochemical and physiological parameters. Integrating mobile health infrastructure with IoT frameworks—networks of interconnected devices that communicate and share data in real-time—will improve access to clinical data, thereby enhancing chronic disease management and preventive care strategies (221).

A growing focus is on developing sustainable and economically viable diagnostic methodologies. Initiatives to reduce environmental impact, such as optimizing reagent use and waste management, align with sustainability goals. Scaled manufacturing processes and optimized resource allocation will improve access to advanced diagnostic platforms. This field’s evolution emphasizes patient-centered care, leveraging technological advancements to provide accessible and personalized healthcare services. Collaboration among research communities, clinical practitioners, and industry partners is crucial for driving innovation, overcoming technological limitations, and ensuring equitable access to diagnostic capabilities across diverse healthcare environments.

### 7.2 Collaborative research and development

The successful integration of innovative technologies in clinical biochemistry depends heavily on extensive interdisciplinary collaboration. Molecular biology, physics, engineering, and clinical medicine professionals must collaborate to develop groundbreaking diagnostic instruments and approaches (222). Academic institutions play a fundamental role in conducting primary research and training for scientists. Industry partners transform this research into market-ready solutions, whereas clinical institutions validate and implement these technologies in real-time. The synergistic relationships among these sectors accelerate technological development and acceptance, ensuring alignment with clinical demands and regulatory standards (223).

### 7.3 Regulatory and ethical considerations

Regulatory obstacles often impede the use of innovative technologies for clinical biochemistry. Regulators are responsible for ensuring the safety and reliability of new diagnostic instruments while keeping pace with rapid technological advancements (224). Streamlining regulations and promoting open dialog can accelerate technology adoption. Ethical considerations, including data privacy and patient consent concerns, are vital in implementing new diagnostic technologies. Protecting patient data and using them for intended purposes is crucial. Furthermore, obtaining informed consent is essential for building trust in healthcare practices, especially when utilizing advanced technologies, such as genomics or quantum computing (225, 226).

### 7.4 Education and training

The rapid integration of new technologies into clinical biochemistry necessitates a comprehensive overview of healthcare professionals’ education and training programs. Clinical biochemists, laboratory technicians, and clinicians must have the knowledge and skills to apply and interpret these emerging technologies [163] effectively. This will involve updating the curriculum to include theory and practice in new analytical methods, data analysis techniques, and ethical considerations (227). Furthermore, future medical and laboratory education systems should focus on integrating innovative technologies to prepare professionals to manage such dynamic technological environments. Hands-on training, simulations, and case studies are crucial to understanding these innovations and their impact on patient care (228). By developing a culture of lifelong learning and aligning educational programs with technological advancements, the healthcare community can optimize patient care and drive innovation.

### 7.5 Creating comprehensive reference intervals

Reference intervals must be established for various populations, including pediatric and geriatric groups. These intervals are crucial for a precise diagnosis and treatment. To ensure that the reference intervals are applicable across different age groups and demographics, initiatives such as CALIPER should be expanded to encompass diverse populations.

### 7.6 Promoting cost-effective solutions

Cost-effective and scalable technologies can make advanced diagnostics more accessible to a broader range of healthcare facilities. These diagnostic tools, which are cost-effective and scalable, connect researchers to real-world solutions, thereby advancing the field of discovery (229).

## 8 Conclusion

Incorporating recent advancements in established technologies and novel tools into clinical biochemistry may aid the early detection and treatment of patients. To successfully integrate these technologies, addressing the present gaps, such as legislative barriers, costs, training requirements, and ethical considerations, is critical. To encourage cost-effective solutions, collaboration must be strengthened, investing in R&D, education and training programs updated, and innovation-driven through these procedures. Continued research in nanotechnology, bioinformatics, and AI will help improve healthcare efficiency and personalization. The use of AI in clinical biochemistry has both advantages and disadvantages that must be considered. AI systems rely on data to learn patterns that characterize optimal care. If the data are biased, then the AI will be. Also, “black boxes” with bad interpretability and clinical accountability concerns hinder AI-driven models. Data manufactured by abusers constructed without consent and AI replacing human expertise risks are worthy of examination before uptake. In the future, researchers should concentrate on building explainable AI models. In addition, improved regulatory frameworks in SDHS will help to ensure effective interdisciplinary engagement.
